# On the Insanity of Children

**Published:** 1859-01-01

**Authors:** 


### Art. IX.—ON THE INSANITY OF CHILDREN.

M. Brierre du Boismont has lately published some remarks upon this subject, in noticing the dissertation of M. le Paulmier.

M. Brierre du Boismont accounts for the comparative exemption of childhood from mental aberration, by the absence of many of the causes so potent in its production in adult life; not that children do not feel acutely, but their sensations are of a fleeting nature, and in this lies their protection. Nevertheless, children who inherit a disposition to mental disease, or who possess a highly-nervous temperament, and who are exposed to favouring circumstances, occasionally manifest undoubted symptoms of the malady.

Haslam, Perfect, Franck, Burrows, and Spurzheim, have given cases of children under eleven years of age. Greding gives an account of a child of eighteen months, which died of marasmus. It was brought into the asylum at Wuldham with its mother (who was insane), and was then scarcely nine months old. It was subject to paroxysmal nervous attacks, which ended either in an indescribable laugh, or in a fit of mania, during which the little creature tore everything she could lay hands upon. Jacobi refers to several cases of insanity in children, in the asylum at Siegburg. Esquirol treated two children—one of eight and another of nine years—and a girl of fourteen, all labouring under mania; he was also consulted about a child of eleven, in which the disease was melancholia.

Marc gives an account of a little girl of eight, who freely admitted that she wished to kill her own mother, her grandmother, and her father. Her object was, to be possessed of their property, and to have an opportunity of indulging her passions. The child was morose, pale, and silent; when spoken to, her answers were very abrupt. Her health was improved by a residence in the country, but on being brought back to town, she became again pale and melancholy. For a long time the cause remained undiscovered; at length it was found that she practised onanism, which she openly avowed, regretting at the same time that she had not the opportunity of indulging her passion with boys.

M. Brierre du Boismont has himself noted four cases, of children of six, seven, and ten years of age, in whom the symptoms of mental disease were manifest; and at present he has under his care a female child of three and a half years old, born of a paralytic father, which shows the strangest caprices; at one time sad and melancholy; again in the most violent fits of rage, without any cause, and not to be appeased. The intelligence of the child is far beyond its years.

The cases of insanity brought under notice by Le Paulmier cannot be said to belong to childhood; his children are young people; for of thirteen examples, three are fourteen, two fifteen, three sixteen, and five seventeen years of age. Before, however, analysing Le Paulmier's work, Brierre du Boismont turns to English, French, and American authors for information on the subject. In Turnham's "Observations and Statistics of Insanity" there is a table of 21,333 cases. Under ten years, eight cases, and from ten to twenty, 1161 cases are noted. According to Turnham, the greatest number of cases of insanity occurs between thirty and forty. In the United States, however, physicians have remarked the disposition to mental disease is stronger between twenty and thirty than between thirty and forty; and this is fairly ascribed to the earlier age at which young men enter the world and engage in business and politics. One of these beardless men of business said to his physician, "I am convinced this kind of life which I lead will drive me mad or kill me; but I must go on." In four American asylums, which contained 2790 patients, 33·73 per cent. were between twenty and thirty, and 24·41 per cent. between thirty and forty years of age.

That the kind of education which the youth in the United States receive has a powerful influence on the development of insanity is proved by Evans and Worthington, in their reports of the Pennsylvania asylums.

Dr. Wigan gives, in his unpublished writings, an account of crimes committed by young people without any object. The age of the youthful malefactors was between sixteen and seventeen for girls, and between seventeen and eighteen for boys. There was this in common, that there had not previously existed the slightest animosity towards the persons against whom they perpetrated outrages. According to Wigan, the greater number of these young people had epistaxis, which, among the females, appeared with the regularity of menstruation. The crimes were generally committed after the temporary cessation of this habitual flux.

Delasiauve and Schnepf have also furnished information on the insanity of early life. The statistics of v. Boutteville exhibit insanity amongst children in no insignificant proportion. The maximum is presented between the ages of thirty and thirty-four. From five to nine, 0·9 per cent.; ten to fourteen, 3·5; from fifteen to nineteen, 20 per cent.

Aubanel and Thorpe observed in the Bicêtre, in the year 1839, eight cases of mania in children, and one of melancholia, from the age of eleven to eighteen years. Mental disease is undoubtedly more frequent in childhood than is generally supposed. Hereditary tendency to disease, and ill-directed education, play an important part in its production.

A writer in the *Revue des Deux Mondes*, for August, 1848, has with much ability accounted for the frequency of insanity in France. Le Paulmier recognises three forms of mania—maniacal excitement (*excitation maniaque*), mania, and incoherent mania. In the first grade of mania the dissociation of ideas is not always recognisable—it nearly resembles the early stage of drunkenness; in the more advanced degree the dissociation of ideas is remarkable; while in the highest it is such, that no longer two sentences, and sometimes not even two parts of one sentence, are connected.

The diagnosis of the mania of children is at times difficult; meningitis may be confounded with it; but in general the headache, the dilatation of the pupils, and the nausea and repeated vomiting, afford means of fixing the line of demarcation. Mania with stupor (*d'une sorte de stupeur exaltique*) approaches closely certain forms of mental alienation which occur after epileptic seizures, and in which the excitement is associated with obtuseness and hallucinations (*obtusion hallucinatoire*). With respect to prognosis, the insanity of early life, according to the observations of Le Paulmier, ends in recovery; however, Delasiauve has made the remark, that a great susceptibility remains, a disposition to a return of the mental disease; and accordingly, that many patients may be found in the wards appropriated to adults, who had formerly been successfully treated in the division assigned to children.

M. Brierre du Boismont concludes his notice of M. le Paulmier's dissertation by giving the result of his own experience. He says, that in a list of forty-two young people in whom the mental disease commenced between fourteen and sixteen years of age, eighteen times was it inherited from their parents.

In by far the greater number of cases, the disease has manifested itself partly under the influence of hereditary predisposition, and partly under the influence of puberty or menstruation. On inquiring from the parents the character of the children, the answer has almost always been, that they were, without any cause, sometimes sad, and at other times wild and ungovernable; they could never apply themselves steadily to work; they had no talent, or if it existed, it only flared up brilliantly for a moment; they would submit themselves to no rules. Some were apathetic, and were not to be excited by emulation; others exhibited a volatility which could not be restrained; many had been subject to spasmodic attacks. The incubation period was often protracted. In eighteen instances recovery took place, but the persons were liable to relapse; there also remained a remarkable strangeness of character, and an inability to assume any fixed position in life. Some afforded insecure evidence of the recovery being permanent. The conclusion is, that though, in a certain number of cases, recovery takes place, the mental alienation of children and young people is a most serious disease—partly from their antecedents, and partly on account of the imperfect developement of the organs. Adducing the foregoing facts in opposition to Le Paulmier's, M. Brierre du Boismont nevertheless accords to the dissertation the meed of his approbation, looking upon it as the production of a thinking mind, and as a proof that themes selected by authors themselves are more productive of fruit than those which are the subjects of prize essays.

